# Supplementary material for: Spiroplasma impairs testes gene expression in Glossina fuscipes fuscipes
Source: bioRxiv. 2025 Sep 25:2025.09.23.678099. Preprint. [Version 1] doi: 10.1101/2025.09.23.678099 (PMC12485850; doi:10.1101/2025.09.23.678099)
Supplement: Supplement 1 [file media-1.pdf]

Spiroplasma impairs testes gene expression in *Glossina fuscipes fuscipes*

Riccardo Piccinno[1], Giulia Fiorenza[1], Francesco Lescai[1], Simone Carpanzano[1], Fabian Gstöttenmayer[3,4], Kiswend-sida M. Dera[4], Anna Cleta Croce[2], Chantel J. de Beer[4], Mariangela Santorsola[1], Giuliano Gasperi[1,2], Federico Forneris[1], Adly M. M. Abd-Alla[4], Serap Aksoy[3], Anna Rodolfa Malacrida[1,*]

Supplementary Material

Table S1 Information of the genomes added to Kraker2 database for the metagenomic analysis.

Accession	Organims	TaxID
GCF_001645765.1	*Candidatus* Mycoplasma haemobos	432608
GCF_902712995.1	*Candidatus* Mycoplasma haemohominis	1494318
GCF_000281235.1	*Candidatus* Mycoplasma haemolamae str. Purdue	1212765
GCF_000319365.1	*Candidatus* Mycoplasma haemominutum 'Birmingham 1'	1116213
GCF_007858515.1	*Mycoplasma anserisalpingitidis*	519450
GCF_000733865.1	*Mycoplasma buteonis*	171280
GCF_000012765.1	*Mycoplasma capricolum subsp. capricolum* ATCC 27343	340047
GCF_024918975.1	*Mycoplasma cottewii*	51364
GCF_000025845.1	*Mycoplasma crocodyli* MP145	512564
GCF_000687815.1	*Mycoplasma elephantis* ATCC *51980*	1408459
GCF_025779955.1	*Mycoplasma enhydrae*	2499220
GCF_000327395.2	*Mycoplasma feriruminatoris*	1179777
GCF_000238995.1	*Mycoplasma haemocanis* str. Illinois	1111676
GCF_000200735.1	*Mycoplasma haemofelis* str. Langford 1	941640
GCF_024722375.1	*Mycoplasma iguanae*	292461
GCF_000518305.1	*Mycoplasma imitans* ATCC 51306	1399794
GCF_000253095.1	*Mycoplasma leachii* 99/014/6	866629
GCF_000622205.1	*Mycoplasma leonicaptivi* ATCC 49890	1448135
GCF_004335975.1	*Mycoplasma marinum*	1937190
GCF_013008635.1	*Mycoplasma miroungigenitalium*	754515
GCF_013008815.1	*Mycoplasma miroungirhinis*	754516
GCF_000023685.1	*Mycoplasma mycoides subsp. capri* str. GM12	436113
GCF_006228185.1	*Mycoplasma nasistruthionis*	353852
GCF_000508245.1	*Mycoplasma ovis* str. Michigan	1415773
GCF_000477415.1	*Mycoplasma parvum* str. Indiana	1403316
GCF_012934855.1	*Mycoplasma phocoenae*	754517
GCF_012934885.1	*Mycoplasma phocoeninasale*	2726117
GCF_017052595.1	*Mycoplasma procyoni*	568784
GCF_900476175.1	*Mycoplasma putrefaciens*	2123
GCF_000702705.1	*Mycoplasma simbae* ATCC 49888	1408469

GCF_003855455.1	*Mycoplasma struthionis*	538220
GCF_000203215.1	*Mycoplasma suis* KI3806	708248
GCF_016925555.1	*Mycoplasma tauri*	547987
GCF_004362335.1	*Mycoplasma testudineum*	244584
GCF_000687795.1	*Mycoplasma testudinis* ATCC 43263	1408471
GCF_004335995.1	*Mycoplasma todarodis*	1937191
GCF_014068355.1	*Mycoplasma tullyi*	1612150
GCF_000277795.1	*Mycoplasma wenyonii* str. Massachusetts	1197325
GCF_000875755.1	*Mycoplasma yeatsii* GM274B	743967
GCF_025486335.1	*Mycoplasma zalophi*	191287
GCF_024742155.1	*Mycoplasma zalophidermidis*	398174
GCF_002290085.1	*Mesoplasma chauliocola*	216427
GCF_002999455.1	*Mesoplasma coleopterae*	324078
GCF_002930145.1	*Mesoplasma corruscae*	216874
GCF_002749675.1	*Mesoplasma entomophilum*	2149
GCF_000008305.1	*Mesoplasma florum* L1	265311
GCF_000701525.1	*Mesoplasma grammopterae* ATCC 49580	1408447
GCF_002441935.1	*Mesoplasma lactucae* ATCC 49193	81460
GCF_002804105.1	*Mesoplasma melaleucae*	81459
GCF_000702725.1	*Mesoplasma photuris* ATCC 49581	1408448
GCF_000518725.1	*Mesoplasma seiffertii* ATCC 49495	1336238
GCF_002843565.1	*Mesoplasma syrphidae*	225999
GCF_002804025.1	*Mesoplasma tabanidae*	219745
GCF_000483165.1	*Acholeplasma multilocale* ATCC 49900	1278299
GCF_002930155.1	*Entomoplasma ellychniae*	2114
GCF_002804205.1	*Entomoplasma freundtii*	74700
GCF_000518285.1	*Williamsoniiplasma lucivorax* ATCC 49196	1399797
GCF_002803985.1	*Williamsoniiplasma luminosum*	214888
GCF_002804005.1	*Williamsoniiplasma somnilux*	215578
GCF_000687735.1	*Acholeplasma equifetale* ATCC 29724	1408415
GCF_017052655.1	*Acholeplasma equirhinis*	555393
GCF_000526235.1	*Acholeplasma granularum* ATCC 19168	1278304
GCF_900660755.1	*Acholeplasma hippikon*	264636
GCF_003385765.1	*Acholeplasma laidlawii*	2148
GCF_025742995.1	*Acholeplasma manati*	591373
GCF_900444665.1	*Acholeplasma oculi*	35623
GCF_025446935.1	*Acholeplasma vituli*	69473
GCF_003363775.1	*Spiroplasma alleghenense*	216931
GCF_000500935.1	*Spiroplasma apis B31*	1276258
GCF_001029245.1	*Spiroplasma atrichopogonis*	1114980
GCF_001281045.1	*Spiroplasma cantharicola*	362837
GCF_008086545.1	*Spiroplasma chinense*	216932
GCF_000400935.1	*Spiroplasma chrysopicola* DF-1	1276227
GCF_001886855.1	*Spiroplasma citri*	2133

GCF_002795265.1	*Spiroplasma clarkii*	2139
GCF_002237575.1	*Spiroplasma corruscae*	216934
GCF_000565175.1	*Spiroplasma culicicola* AES-1	1276246
GCF_000439455.1	*Spiroplasma diminutum* CUAS-1	1276221
GCF_018831625.1	*Spiroplasma* endosymbiont of *'Nebria riversi'*	2792084
GCF_902809815.1	*Spiroplasma* endosymbiont of *Danaus chrysippus*	2691041
GCF_023846195.1	*Spiroplasma* endosymbiont of *Lariophagus distinguendus*	2935082
GCF_003987485.1	*Spiroplasma* endosymbiont of *Megaselia nigra*	2478537
GCF_918697755.1	*Spiroplasma* endosymbiont of *Phyllotreta cruciferae*	2886375
GCF_001029265.1	*Spiroplasma eriocheiris*	315358
GCF_002813555.1	*Spiroplasma floricola* 23-6	1336749
GCF_004379335.1	*Spiroplasma gladiatoris*	2143
GCF_001715535.1	*Spiroplasma helicoides*	216938
GCF_001274875.1	*Spiroplasma kunkelii* CR2-3x	273035
GCF_001267155.1	*Spiroplasma litorale*	216942
GCF_005222125.1	*Spiroplasma melliferum*	2134
GCF_000517365.1	*Spiroplasma mirum* ATCC 29335	838561
GCF_002865545.1	*Spiroplasma monobiae* MQ-1	1336748
GCF_003339775.1	*Spiroplasma phoeniceum* P40	1276259
GCF_021496725.1	*Spiroplasma platyhelix* PALS-1	1276218
GCF_009866525.1	*Spiroplasma poulsonii*	2138
GCF_000565215.1	*Spiroplasma sabaudiense* Ar-1343	1276257
GCF_000400955.1	*Spiroplasma syrphidicola* EA-1	1276229
GCF_009730595.1	*Spiroplasma tabanidicola*	324079
GCF_000439435.1	*Spiroplasma taiwanense* CT-1	1276220
GCF_001262715.1	*Spiroplasma turonicum*	216946
